# Trends in Childhood Poison Exposures and Fatalities: A Retrospective Secondary Data Analysis of the 2009–2019 U.S. National Poison Data System Annual Reports

**DOI:** 10.3390/pediatric13040073

**Published:** 2021-11-15

**Authors:** Hong Li, Teresa Dodd-Butera, Margaret L. Beaman, Molly Broderick Pritty, Thomas E. Heitritter, Richard F. Clark

**Affiliations:** 1 Biobehavioral and Health Research Center, Department of Doctoral Studies, School of Nursing, Azusa Pacific University, Monrovia, CA 91016, USA; 2 Biobehavioral and Health Research Center, Department of Public Health and Doctoral Studies, School of Nursing, Azusa Pacific University, San Diego, CA 92108, USA; tbutera@apu.edu; 3 Department of Nursing, California State University, San Bernardino, CA 92407, USA; margaret.beaman@ymail.com; 4 California Poison Control System, San Diego, CA 92103, USA; mbroderick1@cox.net (M.B.P.); rfclark@health.ucsd.edu (R.F.C.); 5 Pharmacy Informatics, Eisenhower Medical Center, Rancho Mirage, CA 92270, USA; tomhrx@gmail.com; 6 Department of Emergency Medicine, University of California San Diego Medical Center, San Diego, CA 92093, USA

**Keywords:** childhood poison exposures, childhood poison-related fatalities, relative odds of death from poisoning

## Abstract

Despite significant prevention efforts, childhood poison exposures remain a serious public health challenge in the United States. This study aimed to assess annual trends of pharmaceutical vs. non-pharmaceutical poison exposures in the US among children 0–19 years and compare the odds of death by children’s age group. Poison exposure and fatality data were retrospectively extracted from 2009 to 2019 National Poison Data System (NPDS) annual reports for children in all reported age groups. Overall, there was a significant reduction in the annual population-adjusted poison exposures in children (annual percentage change = −2.54%, 95% CI = −3.94% to −1.15%, *p* < 0.01), but not in poisoning-related fatalities. Children 0–5 had similar odds of dying from exposure to non-pharmaceuticals vs. pharmaceuticals. The odds of children 6–12 dying from non-pharmaceuticals vs. pharmaceuticals was 2.38 (95% CI = 1.58, 3.58), χ^2^ = 18.53, *p* < 0.001. In contrast, the odds of children 13–19 dying from pharmaceuticals vs. non-pharmaceuticals was 3.04 (95% CI = 2.51, 3.69), χ^2^ = 141.16, *p* < 0.001. Suicidal intent accounted for 40.63% of pharmaceutical deaths in children 6–12, as well as 48.66% of pharmaceutical and 31.15% of non-pharmaceutical deaths in children 13–19. While a significant decline in overall childhood poison exposures was reported, a decrease in poisoning-related fatalities was not observed. Children in different age groups had contrasting relative odds of death from pharmaceutical and non-pharmaceutical exposures. Among older children, a greater proportion of poisoning-related deaths was due to intentional suicide. These findings provide evidence of age-specific trends in childhood poison exposure risk and directions for future poison prevention efforts and behavioral health partnerships.

## 1. Introduction

Childhood poisoning remains a serious public health challenge in the United States [1,2]. According to the CDC Childhood Injury Report [3], poisoning represented 5% of the fatal and 2% of the non-fatal unintentional injuries among children ages 0–19, with a fatal injury rate of 8 per million children and a non-fatal injury rate of 1680 per million children. Not only do these poisoning-related exposures contribute to loss of life and devastating grief for families, but they may also pose a significant financial impact on communities and society at large. According to the CDC WISQARS, the combined annual medical and work-loss costs associated with unintentional and suicidal poisoning injuries and deaths among children ages 19 and younger accounted for USD 1.65 billion for deaths, USD 494.1 million for hospitalization, and USD 234.1 million for emergency department visits [4].

There is potential for poison exposures during all stages of a child’s life, from infancy through adolescence. Unintentional pharmaceutical poisonings among young children is a common medical issue for emergency departments and poison centers [5,6], causing an estimated number of seven children younger than age six to be seen in emergency departments every hour [7]. Poorly stored non-pharmaceutical household products, such as cosmetics and personal care products, household cleaning substances, foreign bodies or toys [2], and tobacco products also present a danger to young children [8]. A more recent study estimated that nearly one child under age five is sent to the emergency department every 3 h for ingesting cosmetics [9]. For older children such as adolescents, access to prescription and non-prescription medications, as well as illicit drugs, accounted for most of the unintentional and intentional poison exposures [1,10].

To prevent unintentional overdoses, misuse of pharmaceutical substances, and reduce emergency department visits in children, a number of nationwide initiatives were developed and implemented [11,12]. Effective early engineering advancements include the child-resistant packaging of drugs and unit-dose packaging of iron supplements, especially important in younger age groups [13]. Take-back programs are also in existence to safely dispose of medications when prescriptions are completed or outdated [14]. The most promising educational interventions targeting parents include home safety education for safe storage of medicines [15] and cleaning products and accessibility of the telephone number for Poison Control Centers (PCCs) [16].

The design of targeted poison prevention programs for children at various developmental stages requires a detailed investigation of recent national poisoning trends in this population. The purpose of this retrospective secondary data analysis was to detect national trends in poison exposures during childhood (0–19 years) and examine the age-specific odds of children dying from pharmaceutical vs. non-pharmaceutical poison exposures.

## 2. Methods

### 2.1. Study Population, Setting, and Data

For this study, National Poison Data System (NPDS) 2009 through 2019 annual reports served as the principal data source. The NPDS was officially launched by the American Association of Poison Control Center (AAPCC) in 2006, which became the only nationwide comprehensive, near real-time poison exposure surveillance database [17]. Specifically, over a 24-h period every day, healthcare and allied health professionals with specialized toxicology training enter all informational and exposure-related calls received at each of the 55 PCCs into the NPDS [18]. The NPDS products database contains a robust generic coding system that consists of 552 pharmaceutical and 562 non-pharmaceutical groups [19]. The pharmaceutical category includes both licit and illicit drugs. Select examples include analgesics, stimulants/street drugs, antidepressants, and antihistamines. The non-pharmaceutical category includes non-medicinal substances. Select examples in this category include fumes/gas/vapors, cosmetics/personal care products, cleaning substances, and alcohol. See Table 1 for a complete list of major generic substance categories of exposures in the NPDS annual report for 2019. The NPDS publishes annual reports of clinical outcomes and other reported details associated with poison exposures across all age groups. Since 2009, cases of exposures in children have been reported using the following AAPCC age categories: 0–5 years, 6–12 years, and 13–19 years.

The study population comprised poison exposure and fatality cases reported in children younger than 20 years between 2009 and 2019. For each year, demographic and poisoning-related circumstances of childhood fatality cases were retrospectively extracted and categorized by age group (0–5, 6–12, 13–19), type of exposure (pharmaceutical, non-pharmaceutical), and suicidal intent of exposure. According to the NPDS, suicidal attempts were poisoning exposures that were reported to be self-destructive by the callers [19]. For the purpose of this study, fatality case definitions were operationalized using NPDS categories to include those with medical outcome coding of both “death” and “death indirect” reports. In addition, fatality classifications included: “undoubtedly responsible”, “probably responsible” and “contributory”. Adjusted annual rates in poison exposure and fatality per 100,000 children were calculated using population estimates from the U.S. Census Bureau [20,21].

### 2.2. Patient and Public Involvement

Patients were not involved in the design, data extraction, data analysis, or writing of the study.

### 2.3. Statistical Analysis

By using the reporting year as the independent variable, linear regressions were conducted to detect trends in incidence and annual percentage changes (APCs) in poison exposures and fatalities. Independent sample t-tests were used to determine whether the average APC in poison exposures and fatalities among each age group was statistically different from zero. Paired sample t-tests were performed to compare poison exposure and fatality incidences over the years. In addition, relative age group frequencies (i.e., proportions) of fatal exposures by intentional suicide utilizing pharmaceutical and non-pharmaceutical agents were analyzed. Chi-square tests for independence were used to compute the comparative odds of death from non-pharmaceutical vs. pharmaceutical exposures for each of the three children age groups, utilizing aggregate-level data [22]. All descriptive analyses, *t*-tests, regression analyses, and chi-square tests were performed with the IBM SPSS Version 27.0 statistic software package. The two-tailed alpha level for statistical significance was set at 0.05.

## 3. Results

### 3.1. NPDS Reported Childhood Poison Exposures

Between 2009 and 2019, regional US PCCs received 15,005,684 calls regarding poison exposures for all children ages 0–19. This accounted for 61.21% of all human poison exposures reported during the 11-year period. Children ages 0–5 years accounted for 77.64%, children ages 6–12 years accounted for 10.03%, and children ages 13–19 years accounted for 11.99% of all childhood exposures.

The total annual childhood poison exposures reported to poison centers in the US decreased 24.39% from 1,533,387 in 2009 to 1,159,326 in 2019, representing a 22.86% reduction per 100,000 children 0–19 years of age. The 11-year reduction followed a linear trend (*B* = −0.95, *p* < 0.001) with an average population-adjusted APC of −2.54% (95% CI = −3.94% to −1.15%, *p* < 0.01). The APC in poison exposure slowed down significantly (*p* < 0.01) over the years, from −6.24% (year 2009 to 2010) to 0.48% (year 2018 to 2019).

A declining trend in poison exposure was observed among children 0–5 (*B* = −0.97, *p* < 0.001) and children 6–12 (*B* = −0.83, *p* < 0.01). However, an increasing linear trend was observed among children 13–19 (*B* = 0.73, *p* = 0.01). The average APC among children 0–5 years was −3.03% (95% CI = −4.43% to −1.63%, *p* < 0.001). The rates at which reported annual poison exposures changed had gradually declined (*p* < 0.01), from −6.26% to 0.02% for children 0–5 years.

### 3.2. Pharmaceutical Poison Exposures

The total annually reported childhood pharmaceutical poison exposures decreased 23.54%, from 724,760 in 2009 to 554,178 in 2019, or a 21.99% reduction per 100,000 children. The 11-year declining trend was significant (*B* = −0.95, *p* < 0.001), with an average APC of −2.43% (95% CI = −3.95% to −0.92%, *p* < 0.01). The annual reduction rate slowed down significantly (*p* < 0.01), from −6.22% to 1.20%, including 2009 through 2019.

**Figure 1:**
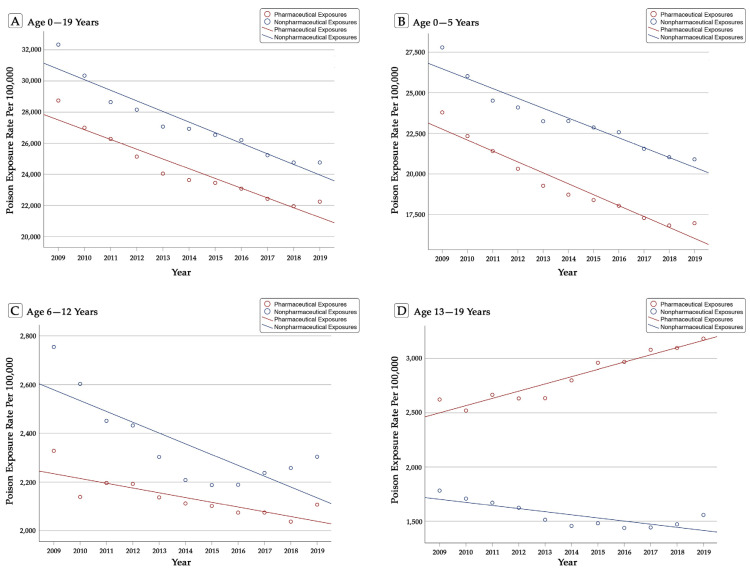
Linear Trends in Population-Adjusted Poison Exposures in U.S. Children, 2009–2019.

For children 0–5 (*B* = −0.97, *p* < 0.001) and 6–12 (*B* = −0.81, *p* < 0.01), a significant downward trend in pharmaceutical poison exposures was noted. However, for children 13–19 (*B* = 0.96, *p* < 0.001), an upward trend in such poison exposures was identified. Over the 11 years, the annual reduction in reported pharmaceutical exposures among children 0–5 years happened at an average APC of −3.31% (95% CI = −4.78% to −1.84%, *p* < 0.001). In this age group, the APCs significantly decreased over the years, from −6.13% to 0.83%, *p* < 0.01. No significant APCs of pharmaceutical exposures were observed among the two older children groups, *p*s > 0.05. See Figure 1 for linear trends in population-adjusted pharmaceutical poison exposures in all children age groups.

### 3.3. Non-Pharmaceutical Poison Exposures

Similarly, the total annually reported childhood exposures to non-pharmaceuticals decreased 25.16% from 808,627 in 2009 to 605,148 in 2019, a 23.65% reduction per 100,000 children. This downward trend followed a linear pattern (*B* = −0.95, *p* < 0.001), with an average APC of −2.64% (95% CI = −4.12% to −1.16%, *p* < 0.01). The declining trend was detected for each age group, *p* < 0.01. Non-pharmaceutical poison exposures decreased at an annual rate of −2.79% (95% CI = −4.35% to −1.22%, *p* < 0.01) among children 0–5 years. See Figure 1 for linear trends in population-adjusted non-pharmaceutical poison exposures in all children age groups.

### 3.4. Non-Pharmaceutical vs. Pharmaceutical Poison Exposures

During the 11-year study period, there was an annual average of 300 more non-pharmaceutical (vs. pharmaceutical) exposures reported per 100,000 children under 20 years, *p* < 0.001. On average, 405 more non-pharmaceutical (vs. pharmaceutical) exposures were reported each year among children 0–5 (*p* < 0.001) and 22 more among children 6–12 (*p* = 0.001), per 100,000 children. In contrast, 127 more pharmaceutical (vs. non-pharmaceutical) exposures were reported each year per 100,000 children 13–19, *p* < 0.001. See Table 2 for 2009–2019 population-adjusted annual rates of poison exposures in children.

### 3.5. NPDS Childhood Poison Exposure-Related Fatalities

The number of poison-related fatalities among children 0–19 increased by 53.16% from 79 in 2009 to 121 in 2019, or 56.16% per 100,000 children. Pharmaceutical fatalities increased 53.85% from 52 to 80, or 57.05% per 100,000 children; non-pharmaceutical fatalities increased 51.85% from 27 to 41, or 54.94% per 100,000 children. Annual poison fatalities increased marginally for children 6–12 (*B* = 0.59, *p* = 0.06) and children 13–19 (*B* = 0.59, *p* = 0.06).

### 3.6. Non-Pharmaceutical vs. Pharmaceutical Poison Fatalities

Overall, there was an annual average of 148 more non-pharmaceutical (vs. pharmaceutical) fatalities reported per one hundred million children under 20 years, *p* < 0.001. An average of 16 more non-pharmaceutical (vs. pharmaceutical) fatalities were reported each year among children 6–12 (*p* < 0.001) per one hundred million children. In contrast, 169 more pharmaceutical (vs. non-pharmaceutical) fatalities were reported each year per one hundred million children 13–19, *p* < 0.001. See Table 3 for 2009–2019 population-adjusted annual rates of poison exposure-related fatalities in children.

### 3.7. Suicidal Intent in Poison-Related Fatalities

No suicidal intent was reported for non-pharmaceutical fatalities in children 0–5 or 6–12. Suicidal intent was reported in 40.63% of pharmaceutical fatalities in children 6–12, as well as in 31.15% of non-pharmaceutical and 48.66% of pharmaceutical fatalities in children 13–19.

### 3.8. Childhood Odds of Death by Poison Exposure and Age

Chi-square tests of independence, utilizing aggregate-level data from the 11 study years, demonstrated similar odds of death from non-pharmaceuticals vs. pharmaceuticals among children 0–5, χ^2^ = 0.87, OR = 0.90 [95% CI = 0.71, 1.13], *p* > 0.05. The odds of children 6–12 dying from non-pharmaceuticals vs. pharmaceuticals was 2.38 (95% CI = 1.58, 3.58), χ^2^ = 18.53, *p* < 0.001. In contrary, the odds of children 13–19 dying from pharmaceuticals vs. non-pharmaceuticals was 3.04 (95% CI = 2.51, 3.69), χ^2^ = 141.16, *p* < 0.001.

## 4. Discussion

Through a retrospective analysis of the 2009 to 2019 NPDS annual reports, the current study examined trends in U.S. poison exposures and related fatalities in children 0–19 years. The findings signal an overall linear decreasing trend in childhood poison exposure to both pharmaceuticals and non-pharmaceutical categories of substances. Of concern, however, is that the number of annually reported fatalities remained largely unchanged for children in all age group categories (0–5, 6–12, and 13–19 years). During the study period, children in age groups of 0–5 and 6–12 years experienced an overall decrease in both types of poison exposures, but children in the 13–19 years age group experienced an increase in pharmaceutical exposures. This increasing rate of pharmaceutical exposures among children 13–19 is consistent with international studies in Australia [23], East Asia [24], and Eastern Europe [25]. Growing numbers of adult prescriptions of analgesic drugs [26], psychoactive drugs [27], and the rising use of non-prescription medications [12] are identified as potential driving factors for this trend. The current study further identified a noteworthy proportion of pharmaceutical exposure-related deaths due to suicidal attempts in age groups of 6–12 and 13–19 years. In the 13–19 years age group, suicidal intent was also reported in non-pharmaceutical fatality cases. Biopsychosocial stressors can be major factors that contribute to suicidal attempts among younger children and adolescents [23,25]. Understanding the environmental, developmental, and biopsychosocial factors across various age groups in children will help to mitigate the risks for unintentional and intentional poison exposures.

Compared to previous findings regarding age-related [28,29] and gender-related [29] patterns for childhood poison exposures, the current study further demonstrated that odds of death by pharmaceuticals vs. non-pharmaceuticals change as children age. Although children 0–5 years were more likely to experience non-pharmaceutical exposures, they had the same odds of death following both types of exposure. Children 6–12 years were more likely to experience non-pharmaceutical exposures and were 138% more likely to die when exposed to non-pharmaceuticals vs. pharmaceuticals. In contrast, children 13–19 were more likely to experience pharmaceutical exposures and were 204% more likely to die, as compared to non-pharmaceutical exposures.

Childhood poison exposures and related injuries and deaths, particularly from suicide attempts, pose a serious public health challenge and take a significant emotional toll on their families and communities [30]. All pediatric healthcare professionals, educators, and caregivers of children must be aware of the developmental risks and the reported age-related odds of death from non-pharmaceutical and pharmaceutical poisons. More specifically, in younger children, increased hand-to-mouth activities, “look-alike” substances, and environmental safety factors should be important targets for poison prevention [6,31,32,33]. In older children and adolescents, safety and educational campaigns should continue to focus on decreasing the risk of unintentional poisonings. Additionally, establishing school-based interventions for suicide attempts [34], considering gender factors [29], and designing age-appropriate health policies [10] are all necessary for addressing intentional exposures in older children and adolescents. Given that school-age children and adolescents are increasingly using the internet and social media platforms to express emotional distress [35] and suicidal ideation [36], there is a critical need to use social networks for the early detection of at-risk populations. Partnering poison centers with social media outlets could be another useful way to expand messaging through prevention initiatives and provide data for identifying trends in at-risk populations. Future explorations researching influences of culture, socioeconomics, and gender along with age-specific associations between exposures and deaths in children could also expand knowledge for prevention strategies and mitigation.

Additionally, future research should investigate risks and protective factors for poison exposures and document individual case-level demographics and health outcome information. Understanding the substance categories most frequently involved in childhood exposures by age group and expanding the trends with a follow-up study will elucidate contributing patterns during the COVID-19 pandemic. Establishing typologies of pharmaceutical and non-pharmaceutical use in self-poisoning in age-specific groups would also be crucial to understanding the extent of variability of substances in suicidal attempts in children [37]. It would also be helpful to delineate prescription vs. non-prescription drugs to see which type and subtypes are more likely to cause fatality among different age groups in children. Future studies to compare specific types of substances, exposure, and pediatric vs. adult poisonings would help to clarify similarities and differences. The information could then be used to shape poison prevention strategies and identify age-related trends beyond childhood.

Overall, community settings such as schools can offer opportunities to disseminate information to students and families, utilizing school nurses [38] and counselors as valued available resources. In addition, given the International Programme on Chemical Safety’s (IPCS) recommendations for promoting and strengthening PCCs [39], using each regional PCC poisoning data and resources in partnership with nearby childcare and healthcare agencies could be an important approach for creating evidence-based prevention efforts. Moreover, an expanded outreach program could be established for increased follow-up with phone consultations to PCC callers. Knowledgeable healthcare providers, such as registered nurses [40] and poison center professionals, would effectively add to the current prevention efforts. Specifically, programs for nurses working with PCCs provide parents an opportunity for individualized assessment of the home environment with highly trusted professionals in the nursing role [38]. These interprofessional collaborative efforts would be key to increasing the impact of prevention programs on poison exposures and ultimately fatalities among all children age groups.

The current study is limited by the reliance on aggregate information from a single data reporting system. The NPDS annual reports do not necessarily represent all exposures and/or poisoning deaths at the national level. Suspected suicide attempts represent what was reported and recorded in the category of “intent”, specified by the NPDS. Exposure reasons of some cases were reported as “undetermined” or “unknown”. The current study also was limited to eleven years of data since specific age groups of childhood poisoning cases have only been available since 2009. Finally, there is increasing usage of the internet and social media platforms for readily available information on topics related to poison exposures [16]. This could be a contributing factor to the declining trend in reported cases in NPDS. Despite these limitations, the findings remain valuable to the literature as it reflects national trends in age-related exposures and fatalities that can contribute to poison prevention efforts among the children population.

## 5. Conclusions

In summary, from 2009 to 2019, the annual number of reported poison exposures in U.S. children 0–19 years of age decreased significantly while the number of reported fatalities remained largely unchanged. There were age-related contrasting odds of death due to exposure to pharmaceuticals vs. non-pharmaceuticals among children. A significant proportion of pharmaceutical fatalities in children 6–12 and 13–19 and non-pharmaceutical fatalities in adolescents was attributed to suicidal intent. Findings of this 11-year retrospective study of childhood poisoning data support policy development and public health practice for reducing the burden of poison-related injury and mortality among children. Addressing both unintentional and intentional childhood poisonings and outcomes are important considerations for targeted and effective poison and injury prevention programs. Exploration of contributing sociodemographic factors of race, ethnicity, and economics could potentially reduce disparities in poisoning risk and promote equity in poison prevention in vulnerable communities. The current research also supports strengthening interprofessional education on age-specific poison prevention efforts for all communities in order to reduce exposures and fatalities in children equitably.

## Figures and Tables

**Table 1:** Major Generic Substance Categories of Exposures in Pharmaceuticals and Non-Pharmaceuticals as listed in 2019 NPDS Annual Report.

Pharmaceuticals (N = 28)	Non-Pharmaceuticals (N = 30)
Analgesics	Adhesives/Glues
Anesthetics	Alcohols
Anticholinergic Drugs	Arts/Crafts/Office Supplies
Anticoagulants	Automotive/Aircraft/Boat Products
Antidepressants	Batteries
Antihistamines	Bites and Envenomations
Antimicrobials	Building and Construction Products
Antineoplastics	Chemicals
Asthma Therapies	Cleaning Substances (Household)
Cardiovascular Drugs	Cosmetics/Personal Care Products
Cold and Cough Preparations	Fumes/Gases/Vapors
Diagnostic Agents	Heavy Metals
Dietary Supplements/Herbals/Homeopathic	Hydrocarbons
Diuretics	Industrial Cleaners
Electrolytes and Minerals	Infectious and ToxinMediated Diseases
Eye/Ear/Nose/Throat Preparations	Information Calls
Gastrointestinal Preparations	Lacrimators
Hormones and Hormone Antagonists	Matches/Fireworks/Explosives
Muscle Relaxants	Miscellaneous Foods
Narcotic Antagonists	Other/Unknown Nondrug Substances
Radiopharmaceuticals	Paints and Stripping Agents
Sedative/Hypnotics/Antipsychotics	Pesticides
Serums, Toxoids, Vaccines	Plants
Stimulants and Street Drugs	Polishes and Waxes
Topical Preparations	Radiation
Unknown Drug	Sporting Equipment
Veterinary Drugs	Swimming Pool/Aquarium
Vitamins	Tobacco/Nicotine/eCigarette Products
	Waterproofers/Sealants
	Weapons of Mass Destruction

**Table 2:** Population-adjusted Annual Rates of Poison Exposures per 100,000, by Type and Children Age Range, 2009–2019.

Year	Pharmaceutical Exposures (per 100,000 Children)			Non-Pharmaceutical Exposures (per 100,000 Children)		
	<6	6 to 12	13 to 19	<6	6 to 12	13 to 19
2019	1696.0	210.7	318.2	2090.3	230.4	155.8
2018	1682.1	203.7	309.6	2103.2	225.7	147.2
2017	1727.9	207.4	307.9	2155.2	223.7	144.3
2016	1803.8	207.4	296.7	2257.3	218.9	143.8
2015	1839.2	210.2	296.0	2286.5	218.8	148.1
2014	1872.3	211.2	279.8	2326.3	220.8	145.7
2013	1927.2	213.7	263.5	2325.0	230.3	151.4
2012	2031.5	219.2	263.3	2409.7	243.2	162.4
2011	2141.4	219.6	266.6	2451.7	245.1	167.1
2010	2233.3	213.9	252.1	2602.8	260.3	170.9
2009	2379.2	232.8	262.2	2779.8	275.4	178.3

**Table 3:** Population-adjusted Annual Rates of Poison Exposure-related Fatalities per 100,000, by Type and Children Age Range, 2009–2019.

Year	Pharmaceutical Exposure-Related Fatalities (per 100 Million Children)			Non-Pharmaceutical Exposure-Related Fatalities (per 100 Million Children)		
	<6	6 to 12	13 to 19	<6	6 to 12	13 to 19
2019	21.2	14.0	241.5	59.3	41.9	51.0
2018	71.5	7.0	323.3	54.7	31.3	23.8
2017	41.9	20.8	245.1	16.7	34.7	40.9
2016	66.8	17.4	126.1	58.4	6.9	34.1
2015	66.8	6.9	184.0	50.1	24.3	44.3
2014	41.9	10.4	180.0	62.8	41.7	47.5
2013	58.4	3.5	226.8	79.3	34.8	37.2
2012	78.8	10.4	239.5	62.2	20.9	33.7
2011	49.6	7.0	190.6	53.7	20.9	33.4
2010	66.0	3.5	174.8	86.6	10.5	46.2
2009	28.8	10.6	137.5	57.6	24.6	19.6

## Data Availability

Data are available upon reasonable request.
